# Frailty predicts trajectories of quality of life over time among British community-dwelling older people

**DOI:** 10.1007/s11136-015-1213-2

**Published:** 2016-01-09

**Authors:** Gotaro Kojima, Steve Iliffe, Richard W. Morris, Yu Taniguchi, Denise Kendrick, Dawn A. Skelton, Tahir Masud, Ann Bowling

**Affiliations:** Department of Primary Care and Population Health, University College London (Royal Free Campus), Rowland Hill Street, London, NW3 2PF UK; School of Social and Community Medicine, University of Bristol, Bristol, UK; Research Team for Social Participation and Community Health, Tokyo Metropolitan Institute of Gerontology, Tokyo, Japan; Division of Primary Care, School of Medicine, University of Nottingham, Nottingham, UK; School of Health and Life Sciences, Institute of Applied Health Research, Glasgow Caledonian University, Glasgow, UK; Department of Health Care for Older People, Nottingham University Hospitals NHS Trust, Nottingham, UK; Faculty of Health Sciences, University of Southampton, Southampton, UK

**Keywords:** Frailty, Quality of life, Well-being, Community-dwelling older people

## Abstract

**Purpose:**

To investigate associations between baseline frailty status and subsequent changes in QOL over time among community-dwelling older people.

**Methods:**

Among 363 community-dwelling older people ≥65 years, frailty was measured using Frailty Index (FI) constructed from 40 deficits at baseline. QOL was measured using Older People’s Quality of Life Questionnaire (OPQOL) six times over 2.5 years. Two-level hierarchical linear models were employed to predict QOL changes over time according to baseline frailty.

**Results:**

At baseline, mean age was 73.1 (range 65–90) and 62.0 % were women. Mean FI was 0.17 (range 0.00–0.66), and mean OPQOL was 130.80 (range 93–163). The hierarchical linear model adjusted for age, gender, ethnicity, education, and enrollment site predicted that those with higher FI at baseline have lower QOL than those with lower FI (regression coefficient = −47.64, *p* < 0.0001) and that QOL changes linearly over time with slopes ranging from 0.80 (FI = 0.00) to −1.15 (FI = 0.66) as the FI increases. A FI of 0.27 is the cutoff point at which improvements in QOL over time change to declines in QOL.

**Conclusions:**

Frailty was associated with lower QOL among British community-dwelling older people. While less frail participants had higher QOL at baseline and QOL improved over time, QOL of frailer participants was lower at baseline and declined.

**Electronic supplementary material:**

The online version of this article (doi:10.1007/s11136-015-1213-2) contains supplementary material, which is available to authorized users.

## Introduction

Frailty in older people is a state characterized by vulnerability to poor resolution of homeostasis as a result of age-related cumulative decline in multiple physiological systems [1]. Frail older people have been shown to be vulnerable to adverse health outcomes, such as falls, hospitalization, disability, and mortality [1–3]. Compared with these outcomes, which have been extensively studied among community-dwelling older people, investigations into the effects of frailty on quality of life (QOL) have only recently commenced and the evidence is relatively limited [4].

Although no consensus has been reached on the definition of frailty, the physical phenotype has been widely used in various research and clinical settings [5]. The phenotype criteria were described in the Cardiovascular Health Study by Fried et al. [2] and consist of five components: unintentional weight loss, self-reported exhaustion, weakness, slow walking speed, and low physical activity, where having 3 or more components is considered as being frail, 1 or 2 as prefrail, and 0 as robust. Another popular approach to operationalize frailty is the Frailty Index (FI). This is a deficit accumulation model to conceptualize frailty by quantifying health deficits accumulated during the life course [6]. The FI is a continuous score ranging from 0 (no deficit) to 1.0 (all deficits present), and the deficits for constructing FI are multidimensional, while Fried’s phenotype mainly focuses on physical components and creates only three categories: frail, prefrail, and robust. Therefore, the FI may be suitable for more precisely capturing the associations with another multidimensional concept, QOL. The FI has been shown to predict mortality more accurately than the phenotype in previous cohort studies [7, 8].

As the proportion of older people has been increasing worldwide, it is of increasing interest and importance to add quality of life to extended years of life [9]. QOL is a multidimensional subjective concept reflecting the individual’s physical health, psychosocial well-being and functioning, independence, control over life, material circumstances, and the external environment [9]. Although several instruments have been developed to measure QOL, they were not originally developed for the elderly population and most of them are based on expert opinions and are not multidimensional. The Older People’s Quality of Life Questionnaire (OPQOL) is a unique instrument in that it has been developed to reflect the views of older people and is able to measure multidimensional aspects of QOL [9, 10].

Most studies on the associations between frailty and QOL have shown that frail older people had significantly lower QOL compared with nonfrail counterparts [11–18]. These findings may intuitively agree with an image of older people whose QOL worsens as they become frailer. However, these studies used a cross-sectional design and therefore cannot explore temporal relationships. Effects of frailty on QOL over time have not been investigated, and therefore, the evidence in the literature is limited. The aim of this study was to investigate associations between baseline frailty status and changes in QOL over time by using repeated-measures analysis among British community-dwelling older people.

## Method

### Design and population

This is a secondary analysis of data from a randomized controlled trial, ProAct65+. This trial was a three-arm parallel design cluster-randomized controlled trial conducted in London and Nottingham/Derby in 2008–2013 to examine the effects of two exercise programs among community-dwelling older people. People aged 65 years and older who were able to walk independently and to participate in group exercise classes were recruited by participating general practices. Those who had three or more falls in the previous year or unstable medical conditions, or who were already reaching the exercise target (150 min of moderate-intensity physical activity per week) were excluded [19, 20].

Written informed consent was obtained from each participant. This trial was approved by Nottingham Research Ethics Committee 2, National Health Service Nottinghamshire County and Westminster, Brent, Harrow, Hounslow, and Barnet & Enfield Primary Care Trusts, and registered in ClinicalTrials.gov (NCT00726531) and ISRCTN (ISRCTN43453770).

A total of 1254 trial participants were randomized to two intervention arms and one usual care arm, and 457 participants were allocated to the usual care arm, the sample for this analysis. The trial methodology and procedures were described in detail elsewhere [19, 20].

Among them, 75 participants who did not have any OPQOL measurement over the study period and 19 participants who had 37 or less deficit variables out of 40 to construct FI were excluded, leaving 363 participants for the final analytic sample for the present study.

### Predictor variable: frailty

The FI was constructed using 40 health deficits at baseline. The deficits can be symptoms, signs, disabilities, and diseases that are biologically sensible, accumulate with age, do not peak too early, and cover a range of systems [21]. The 40 deficits used in this study are shown in supplemental material 1, consisting of 16 physical limitations, including activities of daily living and instrumental activities of daily living, 15 comorbidities, four psychological symptoms, and one deficit each for obesity, polypharmacy, general health, low activity, and pain. Some of the deficits were derived from the 12-item Short-Form Survey and the ConfBal Scale [22, 23], which were conducted as a part of the trial baseline examinations. The deficits were scored as 1 if the deficit was present and 0 if absent, or scored as between 0 and 1 to represent severity of the deficits. The FI can range from 0 (no deficit) to 1 (maximum deficits present). The FI was calculated by adding the scores and dividing by the total number of the deficits available for each participant. Missing deficits were excluded from both numerator and denominator. For example, if a participant had information of 40 deficit variables available and had a total of 10 points, FI was calculated as 10 divided by 40 equals 0.25.

### Outcome variable: QOL

QOL was measured using OPQOL at baseline and five follow-up points of 6, 12, 18, 24, and 30 months. The OPQOL is a QOL instrument consisting of 33 questions over eight dimensions, representing multidimensional aspects of QOL (supplemental material 2):

1. life overall
2. health
3. social relationships
4. independence, control over life, and freedom
5. home and neighborhood
6. psychological and emotional well-being
7. financial circumstances
8. leisure and social activities.

Each question has 5-point Likert scales (“strongly disagree,” “disagree,” “neither agree nor disagree,” “agree,” and “strongly agree”), among which participants were required to choose one. The five options are scored from 1 to 5 with higher scores indicating higher QOL. Total OPQOL score can range from 33 to 165. Only those with complete OPQOL data were included in the analyses. This instrument has been evaluated and validated in community-dwelling multiethnic older people in the UK [10, 24].

### Covariates

Sociodemographic information collected at baseline included age, gender, body mass index (BMI), ethnicity, educational status, numbers of comorbidities and medications, annual household income, and enrollment site. Ethnicity was dichotomized as white (British white, Irish white, and any other white) and nonwhite (the rest). Educational status was dichotomized as school level (primary and secondary school) and above (college, university, or higher).

### Statistical analyses

All statistical analyses were conducted using SAS software (version 9.4, SAS institute, Cary, NC) and based on two-tailed significance with *p* < 0.05 considered statistically significant.

With the 6-wave longitudinal data over 2.5 years, the two-level hierarchical linear model was fitted to predict the changes in QOL over time according to baseline frailty status. This model deals with repeated measurements of QOL nested within each individual and describes the trend with time within individuals (level 1 observation) and heterogeneity in the trend across individuals (level 2 observation). We used the SAS “PROC MIXED” function using the “EMPIRICAL” or so-called sandwich estimator for estimating standard errors of the fixed-effects parameters [25]. All models allowed random intercept and random slope within persons to covary with the “UNSTRUCTURED” covariance structure option. The time variable was coded 0–5 for 0 = baseline, 1 = 6-month, 2 = 12-month, 3 = 18-month, 4 = 24-month, and 5 = 30-month follow-ups. The longitudinal outcome was OPQOL at six time points treated as a time-variant continuous variable. The predictor variable was FI observed at baseline treated as a time-invariant continuous variable. Covariates used for adjustment included age, gender, ethnicity, education, and enrollment site. These covariates were measured at baseline and treated as time invariant, and only age was centered on the mean. Body mass index, income, and number of comorbidities and medications were not used for adjustment because similar components were included in OPQOL, FI, or both. The model specification is as follows:

Level 1 model:

1 $${\text{OPQOL}}_{ti} = \beta_{0i} + \beta_{1i} {\text{ TIME}}_{ti} + u_{ti} + \varepsilon_{ti}$$

Level 2 model:

for the intercept:

2 $$\begin{aligned} \beta_{0i} & = \, \gamma_{00} + \, \gamma_{01} {\text{FI}}_{i} \\ & \quad + \, \gamma_{02} \, {\text{Age}}_{i} \left( {{\text{mean}}-{\text{centered}}} \right) \\ & \quad + \gamma_{03} \,{\text{Gender}}_{i} \\ & \quad + \, \gamma_{04} \,{\text{Ethnicity}}_{i} \\ & \quad+ \, \gamma_{05} \, {\text{Education}}_{i} \\ & \quad + \gamma_{06} \,{\text{Site}}_{i} + u_{0i} \\ \end{aligned}$$

for the slope:

3 $$\beta_{1i} = \, \gamma_{10} + \, \gamma_{11} {\text{FI}}_{i} + u_{1i}$$

In the level 1 model, Eq. (1) describes the within-individual trend of OPQOL. In this equation, the OPQOL of participant *i* at time *t* is modeled as a function of TIME, which represents the baseline and follow-up occasions (TIME = 0, 1, 2, 3, 4, 5). The intercept *β*_0*i*_ is OPQOL for participant *i* at baseline (TIME = 0), and the slope *β*_1*i*_ is the linear change OPQOL for participant *i* per each one unit increment of TIME, i.e., 6 months. The quadratic term was tested but omitted because the quadratic time coefficient was not significant. *u*_*ti*_ and *ε*_*ti*_ are the random between-individuals and within-individual errors for participant *i* at time *t*, respectively, and are assumed to be normally distributed.

In the level 2 model, Eqs. (2) and (3) describe the OPQOL trend across individuals. In Eq. (2), *γ*_00_ is the mean OPQOL for participants who were recruited in Nottingham/Derby with FI of 0.0, mean age, male gender, nonwhite ethnicity, and low education at baseline and *γ*_01_, *γ*_02_, *γ*_03_, *γ*_04_, *γ*_05_, and *γ*_06_ are the coefficients for FI, mean-centered age, gender, ethnicity, education, and enrollment site, respectively, representing the effects on the mean level of OPQOL. In Eq. (3), *γ*_10_ and *γ*_11_ represent the effects on the linear trend of OPQOL change over TIME. *u*_0*i*_ and *u*_1*i*_ are the residual random effects.

## Results

Table 1 shows the baseline characteristics of the 363 participants, including those with only baseline OPQOL and those with baseline and at least one OPQOL. In the entire cohort (*n* = 363), mean age was 73.1 (range 65–90) and 62.0 % were women. The majority of the cohort was white (89.5 %). Mean FI was 0.17 (range 0.00–0.66), and mean OPQOL was 130.80 (range 93–163). On average, participants had approximately two comorbidities and four medications. The number of participants who completed the OPQOL at each time point was 339, 228, 194, 174, 174, and 177. The mean OPQOL at each time point was 130.82 [standard deviation (SD) 13.53], 131.67 (SD 16.00), 134.27 (SD 14.64), 134.79 (SD 14.83), 134.13 (SD 14.78), and 133.85 (SD 14.20). A total of 114, 29, 28, 32, 59, and 101 participants had 1, 2, 3, 4, 5, and 6 OPQOL measurements. Those who only returned a complete OPQOL at baseline, compared with those who also returned complete OPQOL at further follow-up points, were older, more likely to be of nonwhite ethnicity, educated only to primary/secondary level, to have income below £20,000, and to have been recruited in London. They had more comorbidities and medications, lower baseline OPQOL score, and higher baseline frailty.

**Table 1:** Baseline characteristics (*N* = 363)

Variables^a^	Entire cohort^b^ *N* = 363	With only baseline OPQOL *n* = 106	With baseline and ≥1 other OPQOL *n* = 233
Age	73.1 ± 6.2	74.2 + 6.6	72.6 + 6.0
Female	225 (62.0 %)	65 (61.3 %)	146 (62.7 %)
White ethnicity	325 (89.5 %)	91 (85.8 %)	231 (91.4 %)
Body mass index	26.8 ± 5.0	27.6 + 5.3	26.42 + 4.9
Education			
University/college	177 (48.8 %)	49 (46.2 %)	112 (52.4 %)
Primary/secondary	183 (50.4 %)	56 (52.8 %)	110 (47.2 %)
Income			
£20,001+	134 (36.9 %)	38 (35.8 %)	94 (44.3 %)
up to £20,000	185 (51.0 %)	51 (48.1 %)	118 (50.6 %)
Site			
London	160 (44.1 %)	56 (52.8 %)	95 (40.8 %)
Nottingham	203 (55.9 %)	50 (47.2 %)	138 (59.2 %)
Number of comorbidities	2.1 ± 1.6	2.3 + 1.5	2.1 + 1.6
Number of medications	4.0 ± 3.2	4.7 + 3.6	3.7 + 3.0
OPQOL	130.82 ± 13.53	127.2 + 14.0	132.5 + 13.0
Frailty Index	0.17 ± 0.12	0.21 + 0.14	0.16 + 0.12

Some percentages do not sum up to 100 % due to missing value. Twenty-four participants did not have baseline OPQOL

OPQOL: Older People’s Quality of Life Questionnaire

^a^Mean ± standard deviation or *n* (%)

^b^Entire cohort: participants who had at least one OPQOL score at any time point

Table 2 shows the estimated coefficients of unadjusted and fully adjusted hierarchical linear models to predict changes in OPQOL over time.

**Table 2:** Changes in Older People’s Quality of Life score over 2.5 years predicted by baseline Frailty Index

	Unadjusted					Fully adjusted				
	Estimate	Standard error	95 % CI		*p* value	Estimate	Standard error	95 % CI		*p* value
			Lower	Upper				Lower	Upper	
Intercept	138.15	1.12	135.94	140.36	<0.0001	135.57	2.74	130.2	141.0	<0.0001
Time	0.79	0.21	0.38	1.20	0.0002	0.80	0.21	0.38	1.21	0.0002
Frailty Index	−43.06	4.81	−52.50	−33.62	<0.0001	−47.64	5.10	−57.65	−37.64	<0.0001
Frailty Index × time	−2.89	1.22	−5.29	−0.49	0.02	−2.95	1.23	−5.35	−0.54	0.02
Age (mean-centered)						0.18	0.12	−0.05	0.41	0.13
Gender (female)						0.69	1.34	−1.94	3.32	0.61
White						3.56	2.23	−0.81	7.93	0.11
High education						2.29	1.34	−0.33	4.92	0.09
Recruited in London						−3.59	1.38	−6.29	−0.88	0.009

**Fig. 1:**
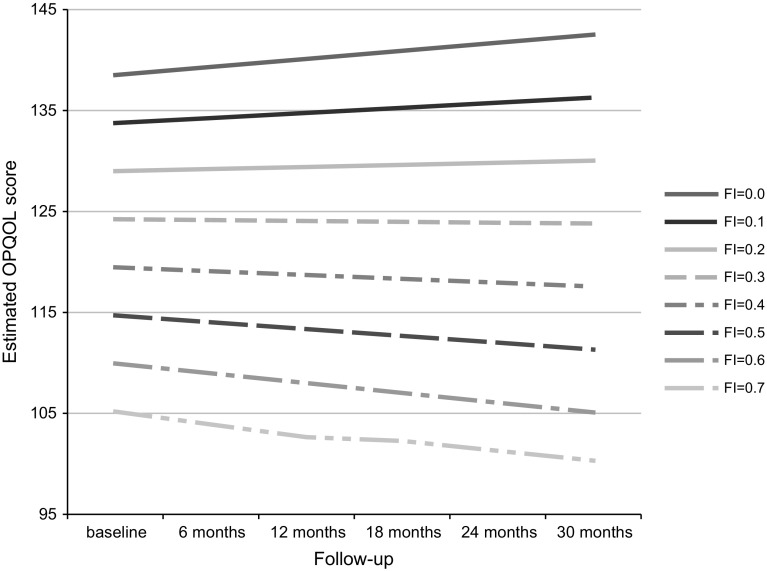
Trajectories of estimated Older People’s Quality of Life score over time by Frailty Index. Estimated by fully adjusted model for participants who were recruited in London with mean age, female gender, white ethnicity, and education above school level. *FI* Frailty Index

In the unadjusted model, the FI has significant negative effects on both OPQOL score (regression coefficient = −43.06, *p* < 0.0001) and change in OPQOL over time (regression coefficient = −2.89, *p* = 0.02). Adding mean-centered age, gender, ethnicity, education, and enrollment site to construct the fully adjusted model has little effect on the associations between the FI and changes in OPQOL over time with similar regression coefficients (−47.64, *p* < 0.0001; −2.95, *p* = 0.02, respectively). Based on these coefficients along with that of “Time” (0.80, *p* = 0.0002), OPQOL change over time (per 6 months) is estimated to be (0.80–2.97 × FI). Frailer participants are predicted to have a lower OPQOL at baseline than those who are less frail. Changes in OPQOL over time also vary with the level of frailty. Those with FI ≤ 0.27 show improvements in OPQOL over time, while those with FI > 0.27 show declines in OPQOL over time, and the rate of decline increases with increasing frailty (Fig. 1).

## Discussion

In this longitudinal study among 363 British older people in the community, we examined changes in QOL over 2.5 years according to baseline frailty status and found that those with greater frailty had a significantly lower baseline QOL. Those with low baseline levels of frailty (FI ≤ 0.27) experienced improvements in QOL over time, while QOL declined over time in those with higher levels of frailty (FI > 0.27). The frailest experienced the fastest declines in QOL over time.

The association between a higher degree of frailty and lower QOL at baseline observed in this study is supported by the findings from the previous cross-sectional studies [11–18]. Across the studies, various frailty definitions (Fried’s phenotype [13–16], the Tilburg Frailty Indicator [11, 12], the Study of Osteoporotic Fractures (SOF) Index [17], and the FI [14, 18]) and QOL instruments (the short-form health survey [11, 13, 15, 16], EUROHIS-8 [11], CASP-19 [18], the Quality of Life Systemic Inventory Questionnaire [14], and OPQOL [17]) were used. Nonetheless, all of these studies demonstrated inverse relationship between frailty and QOL. The single study using OPQOL examined associations of frailty defined by SOF index with QOL and showed that greater frailty was associated with lower QOL based on the total score and for most domains, except for “social relationships and participation” and “financial circumstances” [17].

The only available longitudinal evidence regarding the effects of frailty on QOL in the literature comes from a cohort study of 479 Dutch community-dwelling older people [26]. This study showed significant inverse correlations between frailty defined by the Tilburg Frailty Indicator and subsequent QOL over 1 and 2 years later measured by World Health Organization Quality of Life Questionnaire (WHOQOL-BREF), but failed to adjust for important confounding covariates such as age, gender, ethnicity, socioeconomic status, or education to investigate independent associations.

The study reported here makes a unique contribution to the literature, in its longitudinal design, by adjusting for these confounders and by estimating changes over time at varying levels of frailty. In our study, the least frail participants (FI = 0) were predicted to increase their QOL by 0.80 per 6 months, and the frailest participant (FI = 0.66) was predicted to decrease QOL by 1.15 per 6 months. The cutoff point of the FI corresponding to zero change in QOL over time is approximately 0.27, which is fairly comparable to the cutoff point of FI = 0.25 used to define frailty in the previous studies [8, 27].

Our results should be interpreted with caution. The study participants were originally recruited to and volunteered for the exercise intervention trial. Those who had unstable medical conditions, were at high risk of falling, or were already meeting the recommended target level of physical activity were excluded at the time of the enrollment. Therefore, the participants may be relatively healthier and more motivated to undertake exercise with a higher QOL than general elderly populations. The mean OPQOL of our cohort was 130.8, which is higher than that in previous studies, 108.0–127.0 (calculated to correspond to the 33-item version) of British older people [24]. Although the number and content of health deficits used to create the FI in this study were different from previous studies, the mean FI of the same age group (70–74 years old) of British cohorts in other studies (0.14 [28] and 0.18 [29]) was compatible with ours (0.17). It is noteworthy that, even in this healthier elderly cohort with higher-than-average QOL, QOL is predicted to continue to increase if they remain less frail (FI ≤ 0.27) and decrease if they become frailer (FI > 0.27), independently of age, gender, ethnicity, education, and enrollment site. Those who completed follow-up OPQOL Questionnaires in addition to the baseline OPQOL were more advantaged socioeconomically and were healthier. In particular, their baseline frailty was less and their baseline quality of life was greater. However, the predictors of missing OPQOL follow-up scores are very similar to variables previously identified as related to attrition in the ProAct65+ study [19, 20], and these (apart from comorbidities and medications which are part of the FI definition) have been included in our substantive model. In this case, our inferences assume that follow-up OPQOL scores were missing at random, conditional on these predictors.

This study has multiple strengths. This is, to our knowledge, the first to report changes in QOL over time according to frailty status. QOL was measured using OPQOL, which was, unlike SF-36 or other instruments, originally designed for, and validated with, white British and ethnically diverse older people living in the community in Britain and should yield more reliable data than one measured by other QOL tools. One study compared OPQOL with two other QOL instruments developed for older people, CASP-19 [30] and WHOQOL-OLD [31], for reliability and validity in British population sample based on a random postcode sample and an ethnically diverse population sample [24]. While the other instruments had acceptable levels of reliability and validity only in the former sample, OPQOL did in both the samples [24]. Furthermore, the final model was adjusted for multiple important confounding covariates to assess independent associations between frailty and QOL.

Another prospective cohort study of British community-dwelling older people showed lower QOL at baseline was a significant predictor of incident frailty [32]. In light of our findings that a higher degree of frailty status at baseline predicted declining QOL over time, the relationship between frailty and QOL may be bidirectional. More longitudinal studies are clearly needed to elucidate the associations between frailty and QOL. Moreover, some interventions seem promising to prevent or reverse frailty in older people [1]. Treating frailty may lead to improving QOL and to a better old age.

In conclusion, frailty is associated with lower QOL among British community-dwelling older people. The least frail shows improvements in QOL over time, but frailer older adults experience declining QOL, with fastest declines among the most frail. A cutoff of 0.27 in the FI marks the point at which improvements in QOL over time change to declines in QOL.

## Electronic supplementary material

Below is the link to the electronic supplementary material.

Supplementary material 1 (DOCX 16 kb)

Supplementary material 2 (DOC 114 kb)
