# Supplementary material for: Frailty predicts trajectories of quality of life over time among British community-dwelling older people
Source: Qual Life Res. 2016 Jan 9;25:1743–50. doi: 10.1007/s11136-015-1213-2 (PMC4893360; doi:10.1007/s11136-015-1213-2)
Supplement: Supplementary file 1 — Supplementary material 1 (DOCX 16 kb) [file 11136_2015_1213_MOESM1_ESM.docx]

**Table 1.** List of 40 deficits for constructing frailty index.

|  | variable | grading |
| --- | --- | --- |
|  | **Physical/ADL/IADL limitations (n=16)** |  |
| 1 | Difficulty with public transportation | Present=1, absent=0 |
| 2 | Difficulty with moderate activity | Limited a lot=1, limited a little=0.5, not limited at all=0 |
| 3 | Difficulty with climbing stairs | Limited a lot=1, limited a little=0.5, not limited at all=0 |
| 4 | Difficulty with work activity | Limited a lot=1, limited a little=0.5, not limited at all=0 |
| 5 | Difficulty with sitting in chair | Not confident=1, slightly confident=0.5, confident=0 |
| 6 | Difficulty with getting up of chair | Not confident=1, slightly confident=0.5, confident=0 |
| 7 | Difficulty with picking up something | Not confident=1, slightly confident=0.5, confident=0 |
| 8 | Difficulty with standing unsupported | Not confident=1, slightly confident=0.5, confident=0 |
| 9 | Difficulty with walking indoors | Not confident=1, slightly confident=0.5, confident=0 |
| 10 | Difficulty with walking up slope | Not confident=1, slightly confident=0.5, confident=0 |
| 11 | Difficulty with walking down slope | Not confident=1, slightly confident=0.5, confident=0 |
| 12 | Difficulty with walking over uneven pavement | Not confident=1, slightly confident=0.5, confident=0 |
| 13 | Difficulty with walking down stairs indoors | Not confident=1, slightly confident=0.5, confident=0 |
| 14 | Difficulty with walking up stairs indoors | Not confident=1, slightly confident=0.5, confident=0 |
| 15 | Using walking aids | Yes=1, no=0 |
| 16 | Balance problem | Present=1, absent=0 |
|  | **Comorbidities (n=15)** |  |
| 17 | Respiratory disease | Present=1, absent=0 |
| 18 | Heart/circulatory disease | Present=1, absent=0 |
| 19 | Endocine/metabolic disease | Present=1, absent=0 |
| 20 | Musculoskeletal disease | Present=1, absent=0 |
| 21 | Digestive disease | Present=1, absent=0 |
| 22 | Nervous disease | Present=1, absent=0 |
| 23 | Mental disease | Present=1, absent=0 |
| 24 | Eye disease | Present=1, absent=0 |
| 25 | Genitourinary disease | Present=1, absent=0 |
| 26 | Neoplasms/benign growth disease | Present=1, absent=0 |
| 27 | Infectious disease | Present=1, absent=0 |
| 28 | Ear disease | Present=1, absent=0 |
| 29 | Blood/related disease | Present=1, absent=0 |
| 30 | Skin disease | Present=1, absent=0 |
| 31 | other disease | Present=1, absent=0 |
|  | **Psychological (n=4)** |  |
| 32 | Feeling calm and peaceful | all of the time/most of the time=0, some of the time=0.5, a little of the time/none of the time=1 |
| 33 | Having a lot of energy | all of the time/most of the time=0, some of the time=0.5, a little of the time/none of the time=1 |
| 34 | Feeling downhearted and low | all of the time/most of the time=1, some of the time=0.5, a little of the time/none of the time=0 |
| 35 | Social activity interfered by physical health or emotional problems | all of the time/most of the time=1, some of the time=0.5, a little of the time/none of the time=0 |
|  | **Others (n=5)** |  |
| 36 | Obesity | body mass index >30=1, body mass index <30=0 |
| 37 | Polypharmacy | >6 medications=1, <6 medications=0, |
| 38 | Self-rated general health | Poor/fair=1, good=0.5, very good/excellent=0 |
| 39 | Low activity | no exercise=1, exercise once in a while=0.5, regular exercise=0 |
| 40 | Normal work interfered by Pain | Extremely/quite a bit=1, moderately=0.5, a little bit/not at all=0 |
