# Supplementary material for: Frailty predicts trajectories of quality of life over time among British community-dwelling older people
Source: Qual Life Res. 2016 Jan 9;25:1743–50. doi: 10.1007/s11136-015-1213-2 (PMC4893360; doi:10.1007/s11136-015-1213-2)
Supplement: Supplementary file 2 — Supplementary material 2 (DOC 114 kb) [file 11136_2015_1213_MOESM2_ESM.doc]

**CONFIDENTIAL SERIAL NO.**

**Older People’s Quality of Life Questionnaire (OPQOL-35*)***

**We would like to ask you about your quality of life:**

*Please tick one box in each row. There are no right or wrong answers. Please select the response that best describes you/your views.*

## 1. Thinking about both the good and bad things that make up your quality of life, how would you rate the quality of your life as a whole?

***Y*our quality of life**

**as a whole is:** Very good Good Alright Bad Very bad  **could not**

(1) (2) (3) (4) (5)

**2. Please indicates the extent to which you agree or disagree with each of the**

**following statements.**

*Tick one box in each row*

# Life overall

(1) **I enjoy my life overall** Strongly Agree Neither agree Disagree Strongly

agree or disagree disagree

(1) (2) (3) (4) (5)

(2) **I am happy much of the** Strongly Agree Neither agree Disagree Strongly

**time** agree or disagree disagree

(1) (2) (3) (4) (5)

(3) **I look forward to things**

Strongly Agree Neither agree Disagree Strongly

agree or disagree disagree

(1) (2) (3) (4) (5)

(4) **Life gets me down** Strongly Agree Neither agree Disagree Strongly

agree or disagree disagree

(1) (2) (3) (4) (5)

**Health (wt 4)**

(5) **I have a lot of physical** Strongly Agree Neither agree Disagree Strongly

**energy** agree or disagree disagree

(1) (2) (3) (4) (5)

(6) **Pain affects my** Strongly Agree Neither agree Disagree Strongly

**well-being** agree or disagree disagree

(1) (2) (3) (4) (5)

(7) **My health restricts** Strongly Agree Neither agree Disagree Strongly

**me looking after myself** agree or disagree disagree

**or my home** (1) (2) (3) (4) (5)

(8) **I am healthy enough to** Strongly Agree Neither agree Disagree Strongly

**get out and about** agree or disagree disagree

(1) (2) (3) (4) (5)

**Social relationships (wt 8)**

(9) **My family, friends or** Strongly Agree Neither agree Disagree Strongly

**neighbours would help** agree or disagree disagree

**me if needed** (1) (2) (3) (4) (5)

(10) **I would like more** Strongly Agree Neither agree Disagree Strongly

**companionship or contact** agree or disagree disagree

**with other people** (1) (2) (3) (4) (5)

(11) **I have someone who** Strongly Agree Neither agree Disagree Strongly

**gives me love and affection** agree or disagree disagree

(1) (2) (3) (4) (5)

(12) **I’d like more people to** Strongly Agree Neither agree Disagree Strongly

**enjoy life with** agree or disagree disagree

(1) (2) (3) (4) (5)

**(13) I have my children** Strongly Agree Neither agree Disagree Strongly

**around which is important** agree or disagree disagree/

or no children

(1) (2) (3) (4) (5)

**Independence, control over life, freedom (wt 3)**

(14) **I am healthy enough** Strongly Agree Neither agree Disagree Strongly

**to have my independence** agree or disagree disagree

(1) (2) (3) (4) (5)

(15) **I can please myself**

**what I do** Strongly Agree Neither agree Disagree Strongly

agree or disagree disagree

(1) (2) (3) (4) (5)

(16) **The cost of things** Strongly Agree Neither agree Disagree Strongly

**compared to my pension/** agree or disagree disagree

**income restricts my life** (1) (2) (3) (4) (5)

(17) **I have a lot of control** Strongly Agree Neither agree Disagree Strongly

over the important things agree or disagree disagree

in my life (1) (2) (3) (4) (5)

**Home and neighbourhood (wt 4)**

(18) **I feel safe where I live** Strongly Agree Neither agree Disagree Strongly

agree or disagree disagree

(1) (2) (3) (4) (5)

(19) **The local shops, services** Strongly Agree Neither agree Disagree Strongly

**and facilities are good** agree or disagree disagree

**overall** (1) (2) (3) (4) (5)

(20) **I get pleasure from my** Strongly Agree Neither agree Disagree Strongly

**home** agree or disagree disagree

(1) (2) (3) (4) (5)

(21) **I find my neighbourhood** Strongly Agree Neither agree Disagree Strongly

**friendly** agree or disagree disagree

(1) (2) (3) (4) (5)

**Psychological and emotional well-being (wt 4)**

(22) **I take life as it comes and**

**make the best of things**

Strongly Agree Neither agree Disagree Strongly

agree or disagree disagree

(1) (2) (3) (4) (5)

(23) **I feel lucky compared to** Strongly Agree Neither agree Disagree Strongly

**most people** agree or disagree disagree

(1) (2) (3) (4) (5)

(24) **I tend to look on** Strongly Agree Neither agree Disagree Strongly

**the bright side** agree or disagree disagree

(1) (2) (3) (4) (5)

(25) **If my health limits social/** Strongly Agree Neither agree Disagree Strongly

**leisure activities, then I**  agree or disagree disagree

**will compensate and find** (1) (2) (3) (4) (5)

**something else I can do**

**Financial circumstances (wt 3)**

(26) **I have enough money** Strongly Agree Neither agree Disagree Strongly

**to pay for household bills** agree or disagree disagree

(1) (2) (3) (4) (5)

(27) **I have enough money to** Strongly Agree Neither agree Disagree Strongly

**pay for household repairs** agree or disagree disagree

**or help needed in the house** (1) (2) (3) (4) (5)

(28) **I can afford to buy** Strongly Agree Neither agree Disagree Strongly

**what I want to** agree or disagree disagree

(1) (2) (3) (4) (5)

(29) **I cannot afford to do**  Strongly Agree Neither agree Disagree Strongly

**things I would enjoy** agree or disagree disagree

(1) (2) (3) (4) (5)

**Leisure and activities (wt 6)**

(30) **I have social or leisure** Strongly Agree Neither agree Disagree Strongly

**activities/hobbies that** agree or disagree disagree

**I enjoy doing** (1) (2) (3) (4) (5)

(31) **I try to stay involved** Strongly Agree Neither agree Disagree Strongly

**with things** agree or disagree disagree

(1) (2) (3) (4) (5)

(32) **I do paid or unpaid work** Strongly Agree Neither agree Disagree Strongly

**or** **activities that give me** agree or disagree disagree

**a role in life** (1) (2) (3) (4) (5)

(33) **I have responsibilities** Strongly Agree Neither agree Disagree Strongly

**to others that restrict my** agree or disagree disagree

**social or leisure activities** (1) (2) (3) (4) (5)

(34)  **Religion, belief** Strongly Agree Neither agree Disagree Strongly

**or** **philosophy is important**  agree or disagree disagree

**to my quality of life** (1) (2) (3) (4) (5)

(35) **Cultural/religious** Strongly Agree Neither agree Disagree Strongly

**events/festivals are** agree or disagree disagree

**important to my** (1) (2) (3) (4) (5)

**quality of life**

**Thank you for your help.**

**The links to selected OPQOL documents, questionnaire and scoring can be accessed at no cost in the**

**Archive of International Longevity Centre-UK (ILC-UK) and Economic and Social Research**

**Council (ESRC) websites:**

This is the web link to the ILC publication web page- with links to all the linked publications (e.g. the questionnaire, scoring and norms, etc)

<http://www.ilcuk.org.uk/index.php/publications/publication_details/good_neighbours_measuring_quality_of_life_in_old_age>

This is the direct link to the main report pdf

[www.ilcuk.org.uk/images/uploads/publication-pdfs/pdf_pdf_159.pdf](http://www.ilcuk.org.uk/images/uploads/publication-pdfs/pdf_pdf_159.pdf)

These are the links to the OPQOL questionnaire

<http://www.ilcuk.org.uk/files/pdf_pdf_161.pdf>

[**http**s://www.esrc.ac.**uk**/.../eb39d55e-d234-4f3d-ae30-da5917cb7df6http://www.ilcuk.org.uk/files/pdf_pdf_161.pdf](https://www.esrc.ac.uk/.../eb39d55e-d234-4f3d-ae30-da5917cb7df6http://www.ilcuk.org.uk/files/pdf_pdf_161.pdf).

Older People's Quality of Life Questionnaire

***OPQOL: Copyrighted @ A. Bowling. This questionnaire is free to use and no permissions***

***are needed. The request is that the sources are credited:***

Bowling, A. (2009). Psychometric properties of the Older People’s Quality of Life Questionnaire Validity. **Current Gerontology and Geriatrics Research**. Open access ‘Volume 2009 (2009), Article ID 298950, 12 pages doi:10.1155/2009/298950. [*www.hindawi.com/journals/cggr/2009/298950.abs.htm*](http://www.hindawi.com/journals/cggr/2009/298950.abs.htm)

Bowling A, Stenner P. (2011). Which measure of quality of life performs best in older age? A comparison of the OPQOL, CASP-19 and WHOQOL-OLD. **Journal of Epidemiology and Community Health**, 2011; 65:273-280. **Open access.** doi:10.1136/jech. 2009.087668
